# Development and Validation of a Novel Stenosis Model for Percutaneous Transluminal Angioplasty Training of the Internal Carotid Artery

**DOI:** 10.1007/s00062-025-01545-0

**Published:** 2025-08-21

**Authors:** N. Ramdani, A. A. Kyselyova, N. MacMillan, N. T. Ngo, J. Schmiech, M. Wagner, K. Schulte, D. Krause, F. Flottmann, L. Meyer, M. Bester, J. Fiehler, H. Guerreiro

**Affiliations:** 1 Diagnostic and Interventional Neuroradiology, University Medical Center Hamburg-Eppendorf, Hamburg, Germany; 2 Institute of Product Development and Mechanical Engineering Design, Hamburg University of Technology, Hamburg, Germany; 3 Clinic of Neuroradiology, University Hospital Zurich, Zurich, Switzerland

**Keywords:** Stroke, Endovascular, Intervention, Simulation, Neurology

## Abstract

**Introduction/Background:**

The rising demand for endovascular stroke therapy highlights the need for standardized training environments. Studies have shown the positive impact of simulator-based training for neurointerventional procedures. Given the frequent use of percutaneous transluminal angioplasty (PTA) of the internal carotid artery (ICA) in acute settings, specialized simulation training is crucial. This study evaluates the feasibility of a custom-made ICA stenosis model for PTA simulation.

**Methods:**

Internally developed 3D-printed circular clamps were attached to patient-based 3D printed ICA models and integrated into a previously described realistic whole-body neurovascular simulation model HANNES (Hamburg Anatomic Neurointerventional Endovascular Simulator) to simulate a proximal ICA stenosis. Participants (*N* = 5) of varying experience levels each performed three PTA procedures. Fluoroscopy time, radiation doses, and the extent of stenosis and balloon inflation pressure were assessed. After simulation, participants rated the model in terms of haptic, feasibility and applicability.

**Results:**

For statistical analysis, the participants were divided into two groups according to their experience level. There was a statistically significant difference in the procedure duration between both groups, U = 10.500, Z = −1.968, *p* < 0.05. Significant test results could also be demonstrated for radiation doses (DAP) among groups, Kolmogorov-Smirnov *p* < 0.05, U = 7.000, Z = −2.357, *p* < 0.05. No difference was shown between the total contrast volume (ml) among groups that was used during procedures (m = 11.67, SD 2.09).

**Conclusion:**

The authors propose a novel ICA stenosis simulation model for training of the cervical PTA. The model provides a realistic and replicable method for standardized procedural training.

## Introduction

Stroke continues to be the second biggest cause of death in Europe despite treatment efforts and the increasing number of stroke units throughout the continent. There are about 1.4 million new strokes annually in Europe, and due to increasing life expectancy, the number of incapacitating strokes will likely rise. Considering this, stroke is a pertinent health-related financial issue. For instance, the acute and long-term treatment of strokes cost the European healthcare systems 45 million euros in 2015, either directly or indirectly [1]. About 38% of all ischemic strokes are caused by large artery atherosclerosis, more especially internal carotid artery (ICA) stenosis [2].

Investigators of the CAVATAS study stated that despite the lack of significant differences in the risk of stroke or death, endovascular treatment may be preferable to surgery as it reduces the morbidity related with neck incision and general anesthesia [3]. Endovascular therapy for strokes caused by internal carotid artery dissection has been shown to have high rates of reperfusion and good functional outcomes [4]. Further evidence even shows that carotid artery stenting (CAS) with balloon angioplasty appears to be safe when used as an elective or an emergency intervention. Moreover, at 13 months of follow-up, the rates of restenosis or occlusion for elective and emergency CAS were comparable [5]. The role of CAS vs carotid endarterectomy (CEA) remains controversial. However, the increasing number of patients receiving endovascular treatment in large vessel occlusions (LVO), technical advances in stent design, and in embolic protection devices are likely associated with the ever-increasing interest in CAS. A publication growth rate of > 20% per year on this matter was observed [6]. Nonetheless, it is imperative that clinicians receive adequate training in the procedure.

In order to maintain good outcomes for patients receiving endovascular treatment of ICA, an appropriate operator volume threshold may be twelve or more carotid stent procedures annually (per operator) as several studies indicate [7–9].

It has been demonstrated that technical proficiency and clinical knowledge are interdependent, and that simulated training environments can improve the safe handling and application of medical devices [10]. Three types of training modalities are currently available: synthetic vascular models, computer-based angiography simulators, and animal models [11].

In this study, we integrated a custom-made ICA stenosis model into a previously described neurovascular simulation model [12–14]. Our goal was to evaluate the feasibility of this custom-made ICA stenosis model for simulating percutaneous transluminal angioplasty procedures. We hypothesized that the integration of this stenosis model would successfully replicate key procedural steps, enabling realistic training and experimentation set up within the previously described neurovascular simulation framework.

## Material & Methods

**Fig. 1:**
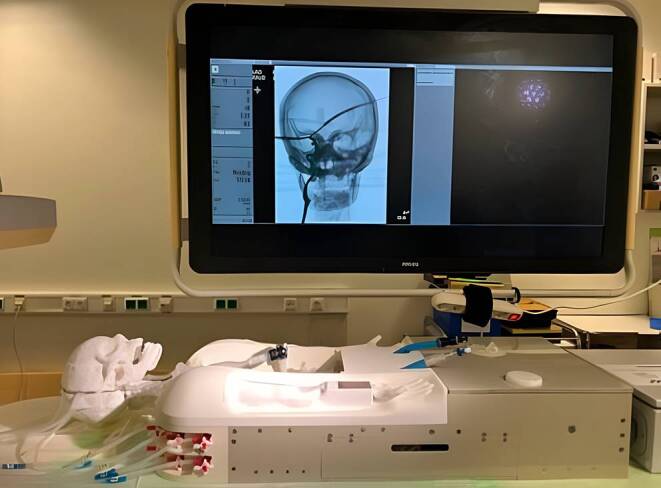
Neurointerventional modular training setup HANNES with its realistic 3D-printed arterial vasculature connected to a flow model. The model is positioned on a procedural table and integrated in an angiography system for simulation and training of neurointerventional procedures. It comprises both femoral and radial accesses as well as a 3D-printed skull model with radiopaque bony landmarks displayed on the depicted cerebral angiogram

Experiments were carried out using HANNES (Hamburg ANatomic Neurointerventional Endovascular Simulator) (Fig. 1), a previously described neurointerventional simulation model, consisting of head, neck, and thorax with corresponding vessels. 3D-printed flexible patient-based neck vessel models comprising a common carotid artery, carotid bifurcation, blind-ended external carotid artery, and internal carotid artery were attached to a rigid skull base prototype [15]. Anonymized patient data and a commercially available 3D printer (Form 2, Formlabs, Somerville, MA, USA) were used for model manufacturing. The simulation model was located in an angiography system (AlluraClarity FD 20, Philips Healthcare, Best, The Netherlands). A physiological environment was created with a water pump that mimics pulsatile blood flow. For this purpose, water was heated to 37 °C with a small amount of baby shampoo to reduce friction.

Angioplasty setup was composed by a short 9F femoral sheath, a 8F Brite tip guide catheter (Cordis, California, USA), a Balance Heavyweight guidewire 0.014 inches 190 cm (Abbott, Illinois, USA) and a Sterling Monorail PTA balloon dilatation catheter 5.5/20 mm (Boston Scientific, Massachusetts, USA).

Participants (*N* = 5) with different levels of experience performed three PTA each (*N* = 15) and were divided into two groups according to their experience level. Group 1 included all participants with an experience level of up to 5 years (2 neurointerventionalists). Group 2 included all participants with an experience level of 5 years or more (3 neurointerventionalists).

**Fig. 2:**
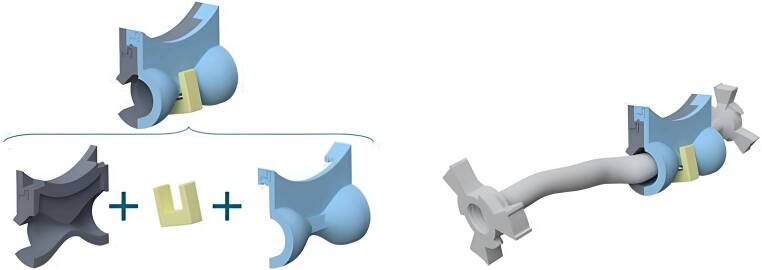
Integration of the 3D-printed carotid model with the stenosis model prototype (on the right) and its three different components: two custom-designed complementary rigid inserts (grey and blue) and one rigid attachment clamp (yellow). The left upper section represents the assembled model, demonstrating the spatial arrangement of its constituent parts. The modular design allows for easy assembly and disassembly, facilitating customization in experimental setups

Participants were blinded to the stenosis model mechanism (Fig. 2). Stenosis was placed in the proximal ICA after the bifurcation.

**Fig. 3:**
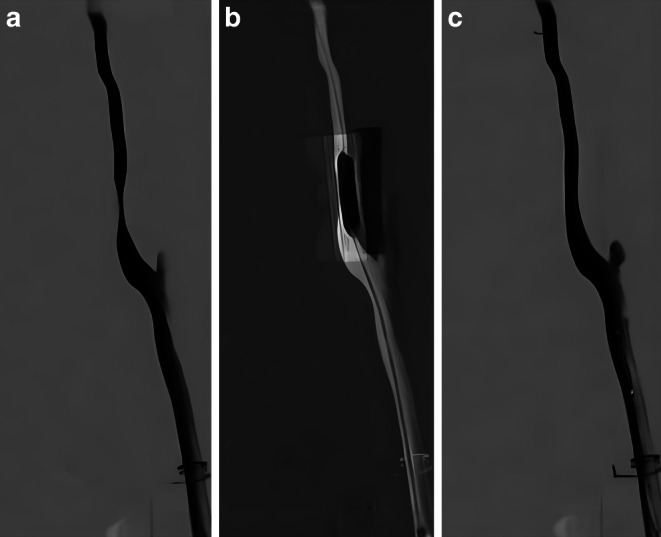
Digital subtraction angiography of the carotid bifurcation, showing a high grade stenosis before the procedure (**a**), during balloon-dilation (**b**) and control angiogram after angioplasty (**c**)

The operators performed a run with iodinated contrast medium (Imeron, Bracco, Milan, Italy) to visualize the stenosis and performed the angioplasty procedure under roadmap guidance (Fig. 3). The procedure involved femoral artery puncture, insertion of a guidewire and catheter, advancement of the catheter to the target vessel under fluoroscopic guidance, contrast injection to visualize the stenosis, balloon angioplasty to dilate the lesion, post-procedural angiographic assessment, and final withdrawal of the catheter system. Procedural duration, fluoroscopy time, radiation doses, stenosis degree and balloon inflation pressure were assessed. After completion of the test series, participants completed a five-point Likert scale questionnaire with 9 questions and options ranging from “strongly disagree” to “strongly agree”, as well as one open-ended question related to possible improvements to the simulator (see supplemental material). Data collection and visualization was performed with Microsoft Excel for Mac (Redmond, WA, USA). Statistical analyses were performed with SPSS 27.0 (IBM, Chicago, IL, USA).

## Results

For statistical analysis, the participants were divided into 2 groups according to their level of experience. Group 1 included all participants with an experience level of up to 5 years (2 subjects, mean = 0.5). Group 2 included all participants with an experience level of 5 years or more (3 subjects, mean = 6.67).

**Fig. 4:**
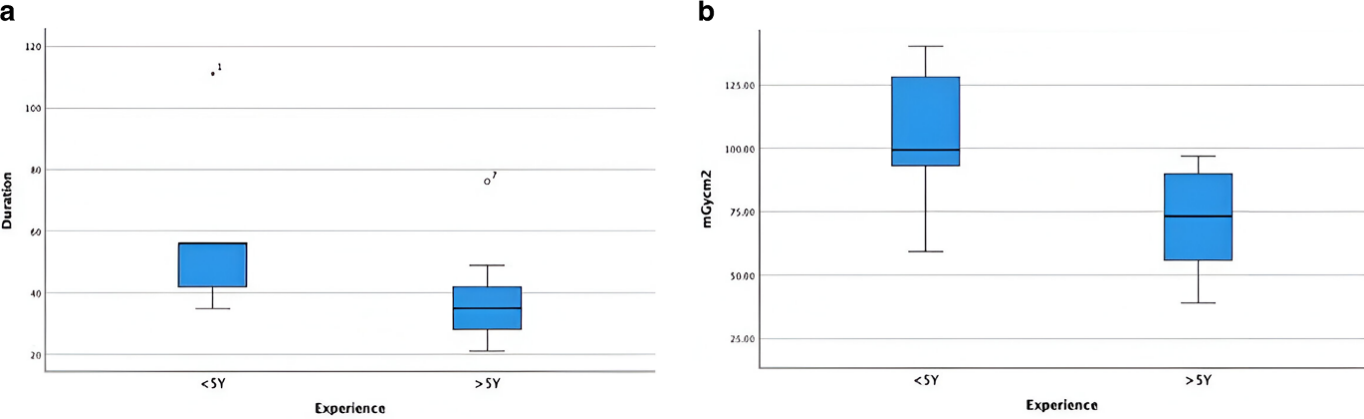
**a** Experience correlated significantly (*p* = 0.05) with procedural duration, **b** Difference in Radiation dose among operators: more experienced physicians (5 or more years of experience) needed significantly (*p* = 0.018) less DAP-radiation dose

A Mann-Whitney-U-Test was calculated to determine the differences in the duration of the procedure between less experienced participants and more experienced participants. The distributions differed between both groups, *p* < 0.05. There was a statistically significant difference in duration between both groups, *U* = 10.500, *Z* = −1.968, *p* < 0.05, mean less experienced group vs. more experienced group (8.5 vs. 5.56) (Fig. 4a).

**Fig. 5:**
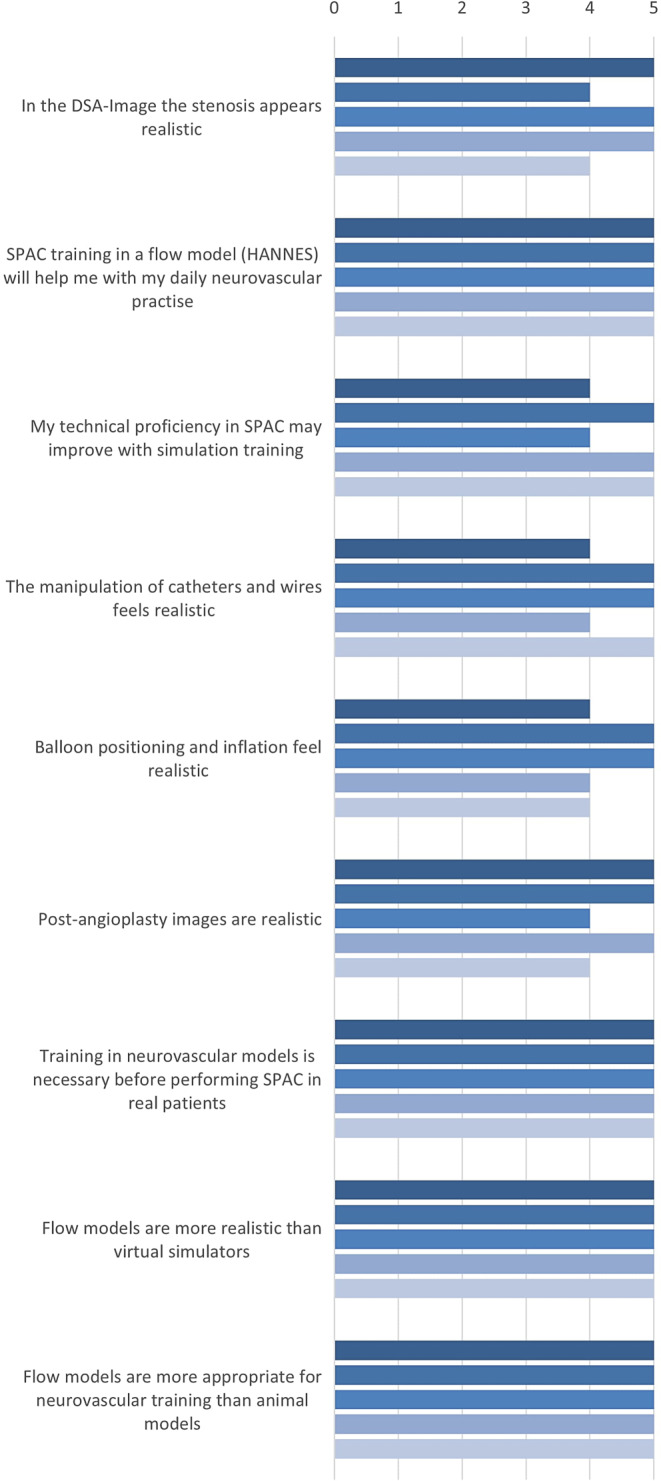
Simulation Feedback Survey

Significant differences could also be demonstrated for radiation doses (DAP) between the two groups, *p* < 0.05, *U* = 7.000, *Z* = −2.357, *p* < 0.05 (Fig. 4b).

No difference was shown between the total contrast volume (ml) among groups that was used during the procedures (mean = 11.67, SD = 2.09).

Balloon inflation pressure was in the upper limit yet considered comparable to in-vivo procedure (13 kPA). Lumen could be normalized in all cases (stenosis mean = 83% before intervention). Participants declared an overall agreement to all statements concerning the beneficial use of the simulation model (Fig. 5). As an open question, it was asked to what extent the simulator could be improved. In this regard, the lack of vasospasm and the option of higher-grade stenosis were noted. Haptics and balloon position were rated as fully realistic (Fig. 5).

## Discussion

In this experimental study-design we aimed to create and validate a stenosis model in a realistic neurointerventional simulation environment. The model offers a standardized procedural training approach that is both realistic and reproducible. We were able to create a high-fidelity training environment with a novel stenosis model and demonstrated its feasibility when integrated in a previously described true-to-life neurovascular simulator. In our testing collective, the more experienced participants presented significantly lower procedural and fluoroscopy times. Lumen normalization and balloon inflation were rated by all the participants as realistic.

The controlled set-up, using real devices together with the capacity to reproduce specific vessel anatomies, with remarkable quality, through additive manufacturing techniques—are two major benefits of this method. This system’s adaptability and modularity allows implementation in a variety of contexts, e.g. in the setting of intracranial stenosis or integrated in an advanced thrombectomy training [16].

The benefit of simulation training in endovascular procedures has been shown in previous works, even in simpler training set-ups [17]. Concerning clinical relevance promising results showed in a prospective, controlled, single center study; patient-specific training before EVAR improved surgical performance (as indicated by fluoroscopy and surgery time and contrast volume used), contributing to an increase in self-confidence of trainees [18].

Currently, most of the endovascular training in the hospital setting is still traditionally performed under the motto “see one, do one” on real patients, which presents a variety of challenges concerning patient safety and ultimately, healthcare costs [19]. Convincingly, criticism of the EVA 3S trial shows the pitfalls of conducting endovascular training and treatment since a significant critique of this study was the fact that CAS was established by comparatively novice interventionalists [20]. Therefore, it is essential to provide structured training programs supported by objective measurements of performance in order to guarantee that only properly qualified individuals do such high-stakes processes [21].

Especially endovascular surgery training is being significantly impacted by a number of factors, including the COVID-19 pandemic-exacerbated reduction in training options and length of residency [22, 23]. Also, vascular surgery has developed into a more autonomous and specialized discipline because endovascular operations are being performed at an increasing rate. Acquiring additional skills is necessary for these relatively new treatment methods. Moreover, research indicates that endovascular operations provide vascular trainees with fewer opportunities for instruction than open surgery, necessitating the use of alternate teaching strategies [24].

To guarantee that vascular residents achieve a high level of performance, a European-wide consensus and regulatory bodies have acknowledged the significance of incorporating simulation into the curriculum and using it as an assessment tool. This approach has been shown to effectively ensure surgical skill proficiency in other specialties [25, 26].

There are limitations that must be noted. The study is based on simulation and was carried out in a flow model with steady physiological parameters, which may not reproduce accurately those observed in patients in an acute setting. This models’ capacity to simulate actual blood flow and reactive vasculature is limited, which makes it challenging to simulate complications like dissection or vessel spasms that may need to be carefully considered during a real procedure. Subsequent research may entail creating in vitro models with the possibility to simulate complications to overcome this constraint. Also, stents were not used in balloon angioplasty for cost reasons.

This study was further limited by the modest number of operators and experiments as well as the participation of experimentees affiliated to one department only. In our model, stenosis is simulated by applying external pressure rather than by reducing the lumen diameter from within (e.g., by plaque formation), which introduces a mechanism that is not entirely realistic.

Multiple factors were described as impacting on types of stenosis, e.g. morphology of the plaque in terms of surface, differences in lipid and necrosis proportion in plaque cores or inflammation [27]. Since our model provides consistently uniform material hardness there might be further investigation in the future regarding different material characteristics. Concerning realistic recreation of complications, thromboembolic events are not currently possible to simulate but further research is ongoing. Prospective experimental set ups could involve manufacturing synthetic plaques, similar to previous development of clot analogs in our model (Guerreiro et al., 2022) [28].
